# Genomic investigation of multispecies and multivariant *bla*_NDM_ outbreak reveals key role of horizontal plasmid transmission

**DOI:** 10.1017/ice.2024.8

**Published:** 2024-06

**Authors:** Nenad Macesic, Adelaide Dennis, Jane Hawkey, Ben Vezina, Jessica A. Wisniewski, Hugh Cottingham, Luke V. Blakeway, Taylor Harshegyi, Katherine Pragastis, Gnei Zweena Badoordeen, Pauline Bass, Andrew J. Stewardson, Amanda Dennison, Denis W. Spelman, Adam W.J. Jenney, Anton Y. Peleg

**Affiliations:** 1 Department of Infectious Diseases, The Alfred Hospital and School of Translational Medicine, Monash University, Melbourne, Australia; 2 Centre to Impact AMR, Monash University, Clayton, Australia; 3 Microbiology Unit, Alfred Hospital, Melbourne, Australia; 4 Infection Program, Monash Biomedicine Discovery Institute, Department of Microbiology, Monash University, Clayton, Australia

## Abstract

**Objectives:**

New Delhi metallo-β-lactamases (NDMs) are major contributors to the spread of carbapenem resistance globally. In Australia, NDMs were previously associated with international travel, but from 2019 we noted increasing incidence of NDM-positive clinical isolates. We investigated the clinical and genomic epidemiology of NDM carriage at a tertiary-care Australian hospital from 2016 to 2021.

**Methods:**

We identified 49 patients with 84 NDM-carrying isolates in an institutional database, and we collected clinical data from electronic medical record. Short- and long-read whole genome sequencing was performed on all isolates. Completed genome assemblies were used to assess the genetic setting of *bla*_NDM_ genes and to compare NDM plasmids.

**Results:**

Of 49 patients, 38 (78%) were identified in 2019–2021 and only 11 (29%) of 38 reported prior travel, compared with 9 (82%) of 11 in 2016–2018 (*P =* .037). In patients with NDM infection, the crude 7-day mortality rate was 0% and the 30-day mortality rate was 14% (2 of 14 patients). NDMs were noted in 41 bacterial strains (ie, species and sequence type combinations). Across 13 plasmid groups, 4 NDM variants were detected: *bla*_NDM-1_, *bla*_NDM-4_, *bla*_NDM-5_, and *bla*_NDM-7_. We noted a change from a diverse NDM plasmid repertoire in 2016–2018 to the emergence of conserved *bla*_NDM-1_ IncN and *bla*_NDM-7_ IncX3 epidemic plasmids, with interstrain spread in 2019–2021. These plasmids were noted in 19 (50%) of 38 patients and 35 (51%) of 68 genomes in 2019–2021.

**Conclusions:**

Increased NDM case numbers were due to local circulation of 2 epidemic plasmids with extensive interstrain transfer. Our findings underscore the challenges of outbreak detection when horizontal transmission of plasmids is the primary mode of spread.

Carbapenem-resistant Gram-negative bacteria cause significant morbidity and mortality globally and were declared a critical priority by the World Health Organization.^1,2^ New Delhi metallo-β-lactamases (NDMs) are significant contributors to the spread of carbapenem resistance.^3^ Although endemic in South and Southeast Asia, NDMs are increasingly noted in other settings, including North America and Europe, where they have caused substantial clonal outbreaks.^4–7^ The spread of NDMs is particularly concerning due to the paucity of treatment options for metallo-β-lactamase infections.^8,9^ Clinical data regarding new treatments, such as cefiderocol and aztreonam-avibactam combinations, are limited, whereas older agents, such as polymyxins, have significant toxicities.^8,9^ Stopping NDM spread is an urgent priority.

In Australia, NDMs have traditionally been associated with travel from endemic countries.^10^ From 2019 onward, we have noted an increasing number of NDM infections and colonization episodes at our institution, which persisted despite coronavirus disease 2019 (COVID-19) travel restrictions. These episodes involved multiple bacterial host species, leading us to hypothesize that local NDM spread may be occurring through horizontal transfer of NDM plasmids. We therefore sought to determine the clinical and genomic epidemiology of NDM carriage at our institution from 2016 to 2021. More specifically, we sought to apply long-read sequencing approaches to characterize NDM genetic settings accurately and to understand within-patient diversity and transfer dynamics of NDM plasmids.

## Methods

### Study setting and population

The study was approved by the Alfred Hospital Ethics Committee. We reviewed an institutional database of carbapenem-resistant Gram-negative isolates spanning 2002–2021 at a healthcare system comprising a tertiary-care hospital, a community hospital, and a geriatric and rehabilitation hospital. We identified NDM carriage in 49 patients (84 isolates) from September 2016 to June 2021, with 81 (96%) of 84 isolates from the tertiary-care hospital and 3 (3.6%) of 84 isolates from the geriatric and rehabilitation hospital. Clinical data were extracted from the electronic medical record (including patient movement data) and a Charlson comorbidity index score calculated.^11^ NDM isolates were classified as associated with colonization or infection.^12^ Crude mortality rates at 7 and 30 days were recorded. Given the study included patients from September 2016 to June 2021, we divided the study into 2 equal periods from first to final NDM patient (2016–2018 and 2019–2021) to allow comparison over time. Patients were screened for carbapenemase-producing organisms following discharge from the intensive care unit (ICU) if they had a history of international hospitalization or were contacts of patients with carbapenemase-producing organisms, according to state guidelines.^13^ Due to the absence of systematic NDM surveillance, we considered that patients may have been colonized in the 30 days prior to first isolation of NDM-carrying organism.^14,15^ We identified overlaps on the same ward at the same time as potential between-patient transmission events.

### Isolate selection and genomic analyses

Routine antimicrobial susceptibility testing was performed using Vitek2 (BioMérieux) to presumptively identify NDM-carrying isolates. Isolates were screened for NDM presence using the Xpert Carba-R multiplex PCR assay (Cepheid) if meropenem MIC was >0.25 mg/L. All 84 NDM-carrying isolates identified were included in the study and underwent short-read (Illumina) and long-read (Oxford Nanopore Technologies) whole-genome sequencing (WGS). One genome was excluded from further analysis due to species mismatch.

Genomic analyses included resistance gene and plasmid replicon detection with Abricate version 1.0.0 software and in silico multilocus sequence typing (MLST) using ‘mlst’ version 2.19.0 software (Supplementary Methods online).^16,17^ We performed core genome-based phylogenetic analyses on key STs (≥2 genomes available from ≥2 patients) to create a core genome alignment and to calculate pairwise single-nucleotide variant (SNV) distances. We identified NDM-carrying contigs that were putative plasmids in long-read assemblies using Abricate. MOB-typer version 1.4.9 software was used to determine plasmid replicons present and to identify plasmid groups according to its clustering algorithm.^18^ We compared individual plasmids using progressiveMauve version 2.4.0.r4736 software,^19^ and we assessed NDM flanking regions using Flanker version 0.1.5 software.^20^

### Statistical analysis

Categorical variables were compared using the χ^2^ test or the Fisher exact test, and continuous variables were compared using the Student *t* test or the Mann–Whitney–Wilcoxon test, as appropriate using R version 4.1.1 software.

## Results

### Study population and clinical characteristics

Of 49 patients with NDM carriage at our institution, 38 (78%) were identified from 2019 to 2021. Clinical characteristics of patients are shown in Table 1. A history of international travel in the prior year was noted in 20 (41%) of 49 patients. Significantly fewer patients reported travel in 2019–2021 than in 2016–2018: 11 (29%) of 38 versus 9 (81%) of 11; *P =* .037). NDM was detected a median of 5 days (IQR, 0–17) after admission. Most patients (36 of 49, 73%) were admitted from the community. Of these 49 patients, 33 (67%) had a history of hospitalization in the 3 months prior, including admission to ICU in 15 (45%) of those 33 patients. The first NDM isolate detected was associated with infection in 7 (14%) of 49 patients. NDM infections developed in 7 (17%) of 42 patients with initial NDM colonization (Table 2). The crude 7- and 30-day mortality rates in patients with NDM infections were 0% and 14% (2 of 14 patients), respectively.

**Table 1.** Clinical Characteristics of Study Cohort

Clinical Characteristic	Total (N=49), No. (%)^a^
**Period of NDM detection**	
2016–2018	11 (22)
2019–2021	38 (78)
**Demographics**	
Age (years), median (IQR)	62 (44–74)
Sex, male	33 (67)
**Comorbidities**	
Pulmonary disease	14 (29)
Diabetes mellitus	11 (22)
Malignancy	9 (18)
Dialysis	5 (10)
Solid organ transplant recipient	4 (8)
Hemopoietic stem-cell transplant recipient	4 (8)
Median Charlson comorbidity index score (IQR)	2 (1–3)
**Risk factors**	
Admitted from community	36 (73)
Admission to hospital in prior three months	33 (67)
Admission to intensive care unit in prior three months	15 (31)
Receipt of antibiotics in prior three months	39 (80)
International travel in prior year	20 (41)
Travel to South Asia	13 (27)
Travel to Southeast Asia	6 (12)
Travel to Middle East	1 (2)
Initial NDM infection	7 (14)
Initial NDM colonization	42 (86)
Subsequent NDM infection	7 (14)

Note. IQR, interquartile range; NDM, New Delhi metallo-β-lactamase.

a Units unless otherwise specified.

**Table 2.** Details of New Delhi Metallo-β-Lactamase (NDM) Infections, 2016–2021

Year of Admission	Days From Admission to NDM	NDM Colonization Status	Site of Infection	Bacterial Host Strain	NDM Variant	NDM Genetic Setting	Prior Travel	Antimicrobial Treatment Regimen	Crude 7-Day Mortality	Crude 30-day Mortality
2016	0	No prior NDM colonization	Urinary tract	*Escherichia coli* ST405	*bla*_NDM-5_	IncFIA (AA170 AH818)	South Asia	Amikacin/ Fosfomycin	No	No
2016	14	Prior NDM colonization	Prostatitis	*E. coli* ST410	*bla*_NDM-5_	IncFIA (AA323 AI214)	South Asia	Tigecycline/ Meropenem/ Fosfomycin	No	No
2018	8	Prior NDM colonisation	Urinary tract	*E. coli* ST405	*bla*_NDM-5_	IncI-γ/K1	South Asia	Tigecycline/ Fosfomycin	No	No
2019	17	Prior NDM colonization	Pneumonia with bacteremia	*Klebsiella pneumoniae* ST15	*bla*_NDM-4_	IncFII (AA450 AI539)	Southeast Asia	Ceftazidime/ Avibactam/ Aztreonam	No	Yes
2019	4	No prior NDM colonization	Wound	*Acinetobacter baumannii* ST2	*bla*_NDM-1_	Chromosome	South Asia	Trimethoprim/ Sulphamethoxazole	No	No
2019	69	Prior NDM colonization	Sternal wound infection	*Klebsiella variicola* ST596	*bla*_NDM-1_	IncN	N/A	Ceftazidime/ Avibactam/ Aztreonam/ Tigecycline	No	Yes
2020	0	No prior NDM colonization	Wound	*K. pneumoniae* ST16	*bla*_NDM-1_	IncFIB (AA405 AI436)	Middle East	Tigecycline	No	No
2020	13	No prior NDM colonization	Wound	*Pseudomonas aeruginosa* ST644	*bla*_NDM-1_	Chromosome	N/A	Colistin/ Meropenem	No	No
2020	44	Prior NDM colonization	Pneumonia	*Klebsiella quasipneumoniae* ST5551	*bla*_NDM-1_	IncN	N/A	Fosfomycin	No	No
2020	9	No prior NDM colonization	Peritonitis	*K. quasipneumoniae* ST5551	*bla*_NDM-1_	IncN	N/A	Tigecycline	No	No
2020	3	Prior NDM colonization	Urinary tract	*Enterobacter hormaechei* ST1015	*bla*_NDM-1_	IncC	N/A	Fosfomycin/ Nitrofurantoin/ Ciprofloxacin	No	No
2020	46	No prior NDM colonization	Wound	*Citrobacter braakii* ST567	*bla*_NDM-7_	IncX3	N/A	Trimethoprim/ Sulfamethoxazole	No	No
2021	138	No prior NDM colonisation	Pleural fluid	*E. coli* ST127	*bla*_NDM-7_	IncX3	N/A	Ciprofloxacin	No	No
2021	17	Prior NDM colonization	Urinary tract	*K. pneumoniae* ST485	*bla*_NDM-1_	IncN	N/A	Trimethoprim/ Sulfamethoxazole	No	No

### Isolate characteristics and genomic epidemiology

**Figure 1.**
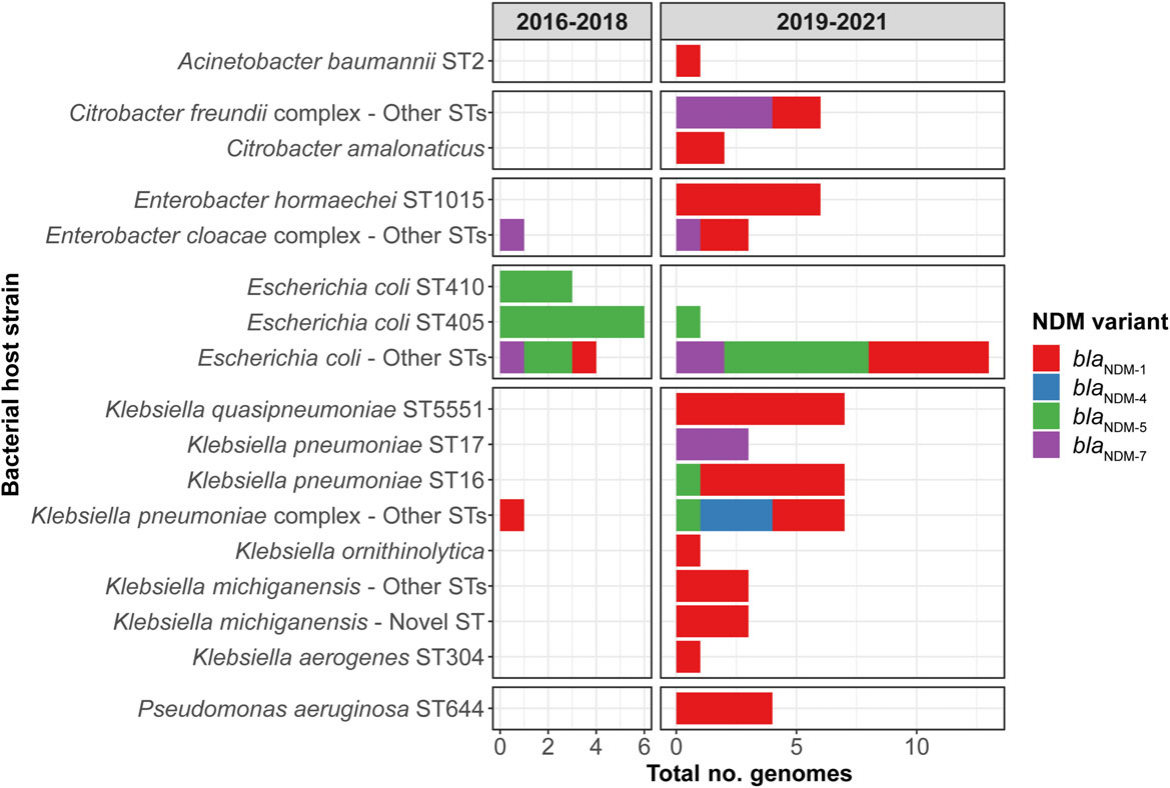
New Delhi metallo-β-lactamase variants and bacterial host strains, 2016–2021. NDM variants and bacterial host strains shown over 2 study periods. There was a diversity in both the bacterial hosts and NDM variants, with 4 NDM variants being noted across 41 bacterial host strains, and a shift from NDM-5 and *Escherichia coli* in 2016–2018 to NDM-1/NDM-7 and non–*E. coli* species in 2019–2021. Note. NDM, New Delhi metallo-β-lactamase; ST, sequence type.

NDM-carrying bacteria were highly diverse, with 41 bacterial strains (defined as unique species/MLST combinations) noted (Fig. 1, Supplementary Table 1 online, and Supplementary Fig. 1 online). No dominant bacterial strain was identified; a maximum of 7 genomes was noted in *Escherichia coli* ST405 (5 patients), *Klebsiella pneumoniae* ST16 (4 patients), and *Klebsiella quasipneumoniae* ST5551 (3 patients). Of 12 strains found in ≥2 patients, 6 strains had between-patient pairwise SNV distances <20 SNVs, suggestive of possible clonal spread (Supplementary Table 2 online). Also, 4 NDM variants were detected: *bla*_NDM-1_ (48 of 83 genomes, 58%), *bla*_NDM-5_ (20 of 83, 24%), *bla*_NDM-7_ (12 of 83, 14%), and *bla*_NDM-4_ (3 of 83, 4%). Non-NDM carbapenemase genes were detected in 11 (13%) of 83 genomes (*bla*_IMP-4_, 4 genomes; *bla*_IMP-62_, 3 genomes; *bla*_OXA-23_-like, 3 genomes; and *bla*_OXA-48_-like, 2 genomes). Also, 10 (12%) of 83 genomes carried *mcr-9.1*, a novel determinant of colistin resistance. We noted changes in epidemiology from *E. coli* in 2016–2018 (13 of 15 genomes, 86%) to non–*E. coli* species in 2019–2021 (54 of 68 genomes, 79%; *P <* .001), and from *bla*_NDM-5_ (11 of 15 genomes, 73%) to non-*bla*_NDM-5_ variants (59 of 68 genomes, 87%; *P <* .001).

### Plasmid analyses

**Figure 2.**
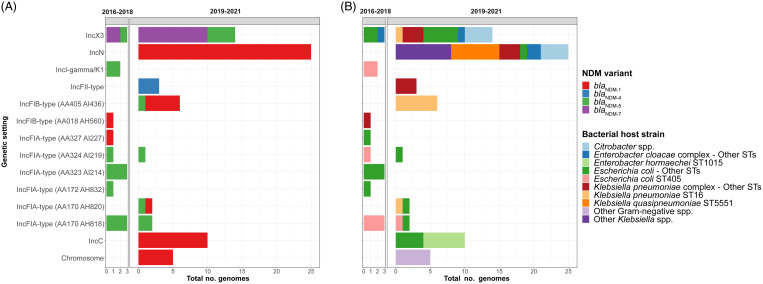
Genetic settings of *bla*_NDM_, 2016–2021. (A) Genetic settings of *bla*_NDM_ and corresponding NDM variants over study, as defined by 2 study periods. We detected NDM variants in 13 distinct plasmid groups as well as integration into the bacterial chromosome. (B) Genetic settings of *bla*_NDM_ and corresponding bacterial host strains over the study, as defined by 2 study periods. Note. NDM, New Delhi metallo-β-lactamase; ST, sequence type.

To assess the hypothesis of plasmid transmission and to identify temporal shifts, we performed plasmid clustering to identify plasmid groups; we quantified SNVs across the plasmid backbone; and we assessed large-scale rearrangements. We detected 13 distinct plasmid groups, of which 3 of 13 had multiple NDM variants present (Fig. 2A), and 8 of 13 were noted in multiple bacterial host strains (Fig. 2B). In 2016–2018, there was a diversity of plasmid groups (8 groups across 11 patients). In 2019–2021, 3 dominant epidemic plasmid groups (*bla*_NDM-1_ IncN, *bla*_NDM-5/NDM-7_ IncX3 and *bla*_NDM-1_ IncC) were noted in 30 (79%) of 38 patients and 49 (71%) of 68 genomes in that period (Fig. 2A).

**Figure 3.**
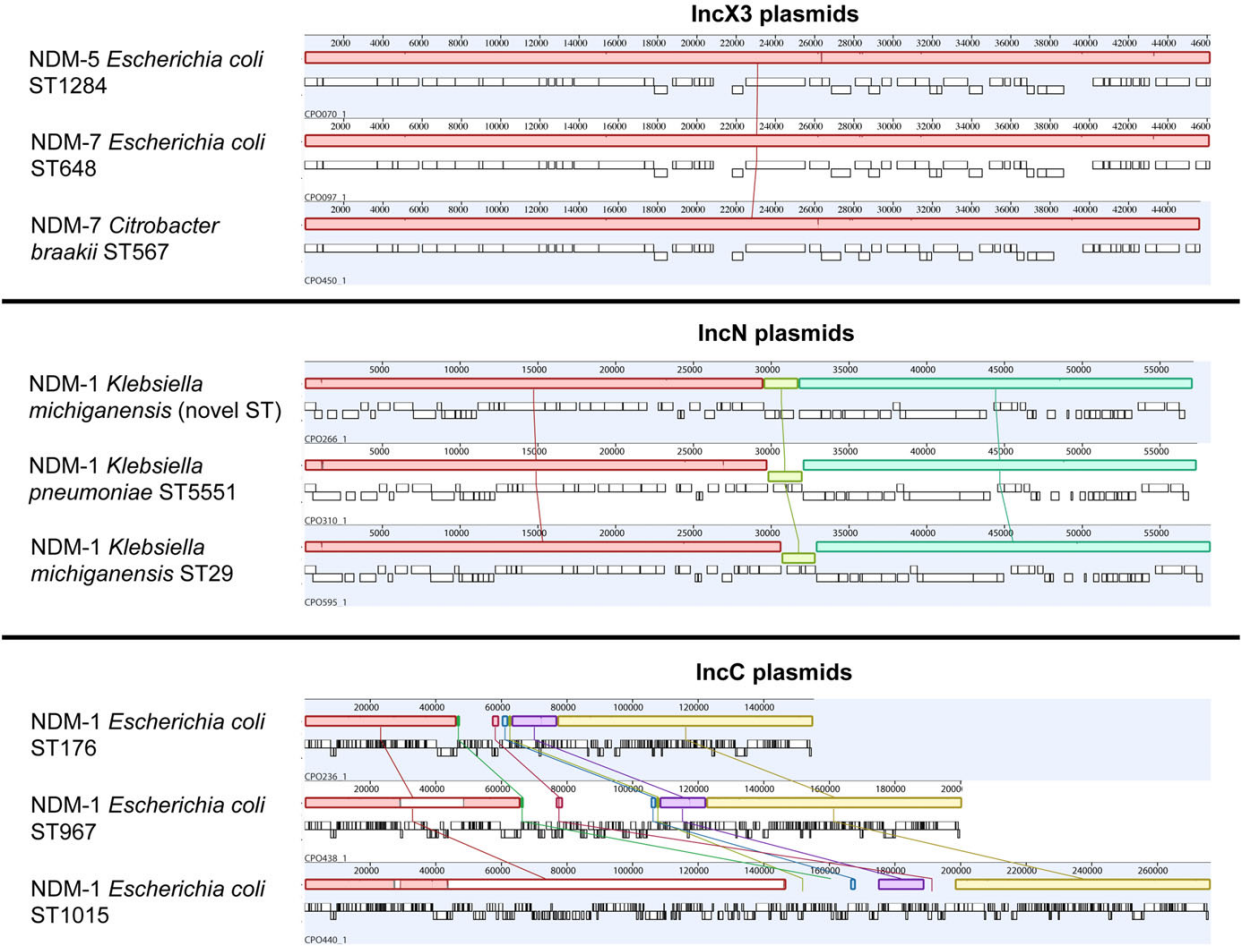
Comparative analyses of NDM IncX3, IncN and IncC plasmids. We aligned representative plasmids from each of the 3 epidemic NDM plasmid groups from our study. Each colored field represents a locally collinear block, a homologous region of sequence shared by multiple plasmids without any rearrangement of that region. The same colors indicate the same regions present in different plasmids. IncX3 plasmids were structurally nearly identical regardless of NDM variant. *bla*_NDM-1_ IncN plasmids had minor structural variation near *bla*_NDM-1_ due to a palindromic sequence. Although they belonged to the same plasmid group, IncC plasmids were more diverse with three plasmid subtypes, as shown. Note. NDM, New Delhi metallo-β-lactamase; ST, sequence type.

Overall, *bla*_NDM-1_ IncN plasmids were the largest group (25 genomes in 14 patients) and were highly homogenous (1 SNV across the backbone and minor structural variation near *bla*_NDM-1_) (Fig. 3 and Supplementary Table 3 online). IncX3 plasmids were similarly homogenous with near identical structures regardless of NDM variant (Fig. 3). Epidemic *bla*_NDM-7_ IncX3 plasmids carried only 2 SNVs across the backbone. Despite being clustered to the same plasmid group, IncC plasmids were more diverse with 23 SNVs across the backbone with an obvious subcluster of 6 near-identical plasmids from 4 patients (median pairwise SNV distance, 1). This subcluster corresponded to a plasmid initially found in *E. coli* ST176 in 2019, then in clonal *Enterobacter hormaechei* ST1015 in 3 other patients in 2020–2021. We compared these epidemic plasmids to plasmids circulating globally (Supplementary Fig. 2 online and Supplementary Table 4 online). IncX3 plasmids were almost identical to global NDM IncX3 plasmids, with 100% coverage and 99.96% identity. IncN and IncC plasmids were divergent to NDM global plasmids. IncN had 95% coverage and 99.93% identity, and IncC had 93% coverage and 99.99% identity.

**Figure 4.**
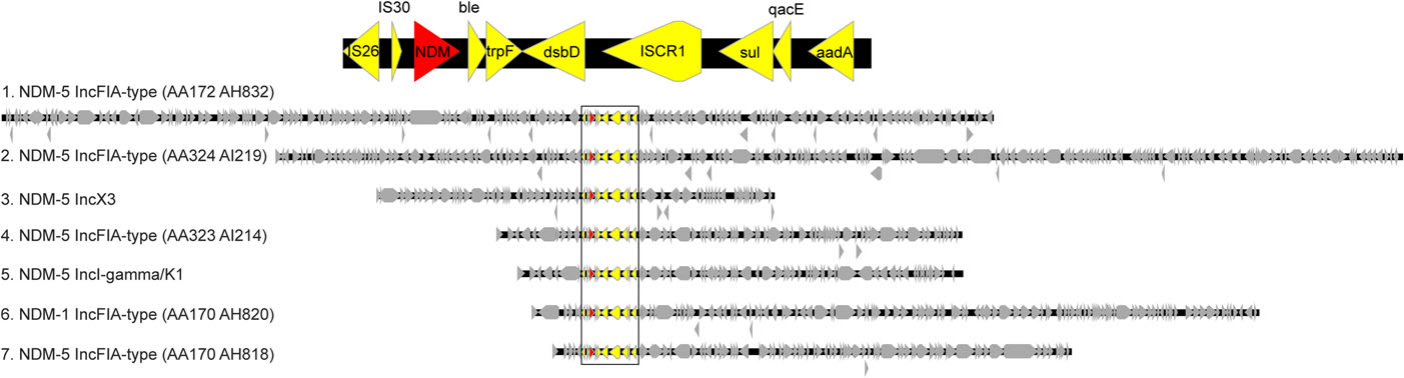
Promiscuous NDM transposon found in 7 plasmid groups. The top of the figure shows the genes contained within the transposon, including *bla*_NDM_. The bottom of the figure shows the transposon (in color) inserting into diverse plasmids from 7 different plasmid groups. This finding was suggestive of movement of the transposon between different plasmid backbones. Note. NDM, New Delhi metallo-β-lactamase.

Other plasmid groups with multiple plasmids available for comparison showed larger-scale structural differences despite sharing a similar backbone (Supplementary Fig. 3 online). Each NDM flanking region was associated with a single plasmid group, except for a transposon capable of carrying 2 NDM variants (*bla*_NDM-1_ and *bla*_NDM-5_) that was identified across 7 plasmid groups (Fig. 4). All patients but 1 with this flanking region had a history of travel to South or Southeast Asia. In 1 patient, we noted both the *bla*_NDM-5_ IncX3 and IncFIA-type (AA170 AH820) plasmids were carrying this flanking region (Fig. 4, plasmids 3 and 6, and Supplementary Fig. 4 online, patient 4). This transposon was not found in any other IncX3 plasmids in the study, which raises the possibility of movement of a transposable element between different plasmids within the patient.

### Within-patient plasmid analysis

**Figure 5.**
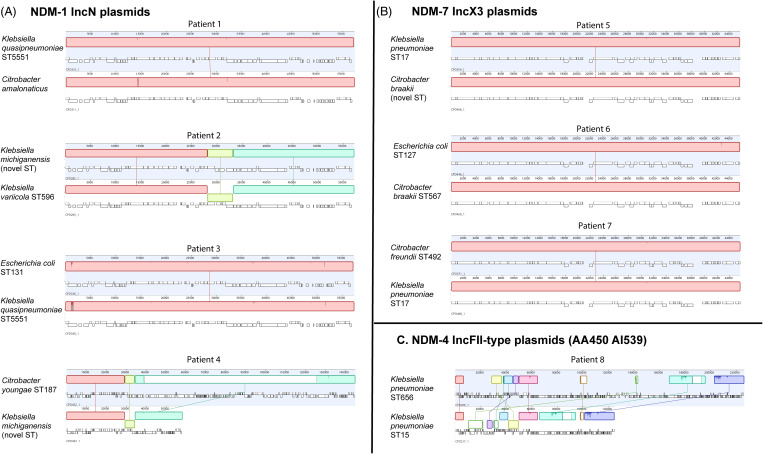
Analysis of potential within-patient plasmid transfer between bacterial host strains. Overall, 8 patients had the same NDM variant/plasmid group combinations across multiple bacterial host strains. We aligned plasmids from each of the bacterial host strains. (A) NDM-1 IncN plasmids. (B) NDM-7 IncX3 plasmids. (C) NDM-4 IncFII-type (AA450 AI539) plasmids. The bacterial host strains are shown on the left of the plasmids. Each colored field represents a locally collinear block, a homologous region of sequence shared by multiple plasmids without any rearrangement of that region. The same colors indicate the same regions present in different plasmids. Note. NDM, New Delhi metallo-β-lactamase; ST, sequence type.

Multiple genomes were available for analysis in 18 (37%) of 49 patients. Moreover, 6 patients had persistent strain colonization (multiple isolates of the same bacterial strain with same NDM plasmid) and 4 patients had multiple colonization events (presence of different plasmid groups), including 2 patients with plasmids carrying different NDM variants (Supplementary Fig. 4 online). In the remaining 8 of 18 patients, we detected the same plasmid group across different bacterial hosts. We compared plasmids within patients to distinguish potential interstrain plasmid transfer from multiple colonization events. The 7 of 8 patients colonized with *bla*_NDM-1_ IncN and *bla*_NDM-7_ IncX3, epidemic plasmids had highly similar plasmids suggestive of interspecies transfer (Fig. 5A and 5B). For the patient colonized with nonepidemic plasmids (Fig. 5C), there were significant structural rearrangements of an IncFII-type plasmid, making it less likely that interstrain plasmid transfer had occurred. In patients with multiple plasmids of the same plasmid group there was a median SNV distance of 0 (range, 0–267), which is shown by plasmid group in Supplementary Table 5 (online).

### Transmission analysis

We analyzed potential in-hospital transmission events by combining detailed patient movement data with plasmid-level genomic data. We defined a potential transmission event when there was spatiotemporal overlap between patients that shared closely related NDM plasmids (ie, the same plasmid group and NDM variant with ≤5 SNVs difference in the backbone). Despite multiple patients having highly similar plasmids, only 8 patients were linked in 5 potential transmission events, all involving the IncN plasmid (Supplementary Fig. 5 online). Despite using criteria aimed at increasing sensitivity, we were unable to directly link the cases of 41 (84%) of 49 patients.

## Discussion

In this study, we combined clinical data with insights from long-read genomic sequencing to analyze the epidemiology of NDM-carrying bacteria at our institution. Although reports of previous NDM outbreaks noted clonal spread of specific strains such as *K. pneumoniae* ST147 and ST307,^5,6,21^ increased case numbers in our institution were due to circulation of several epidemic plasmids. For most patients, we were unable to document clear healthcare-associated NDM acquisition, highlighting the possibility of acquisition in the community or through undetected sources in the hospital. Ultimately, 29% patients developed NDM infection and the 30-day crude mortality rate among these patients was 14%, consistent with previously reported mortality rates of ∼10%–20%.^22,23^ Our findings also underscore the challenges of outbreak detection when horizontal transmission of plasmids is the primary mode of spread, particularly due to the remarkable plasticity of plasmids, which can undergo substantial changes even within a single patient.^24^

We noted significant shifts in NDM epidemiology during the study. From 2016 to 2018, most colonized patients had a history of travel to endemic regions such as South and Southeast Asia, as noted previously in our setting.^10^ Correspondingly, both the bacterial hosts and plasmids carrying NDMs were diverse with a predominance of *E. coli* and *bla*_NDM-5_, and no transmission events between patients were detected. This finding contrasted with 2019–2021, when we noted increased NDM case numbers driven by patients with no travel history, which persisted despite strict travel restrictions imposed in Australia due to the COVID-19 pandemic.

Rather than being due to a single bacterial clone or plasmid, this spread was due to IncN and IncX3 epidemic plasmids. These plasmids were capable of extensive interstrain transfer, found in 14 and 11 bacterial strains, respectively. IncX3 plasmids have been recognized as drivers of NDM spread globally^3,25,26^ and plasmids from our study closely matched internationally circulating plasmids. NDM IncN plasmids have previously been less recognized.^27–30^ In our study, both plasmids were highly conserved across different bacterial hosts and different patients, suggesting circulation of these plasmids and acquisition by multiple patients. We also noted multiple NDM IncC plasmids; however, these were more heterogenous, comprising a subcluster of 6 highly related plasmids across 4 patients that suggested horizontal transfer of the IncC plasmid then clonal spread of *E. hormaechei* ST1015.

Given these genomic findings, we attempted to detect healthcare-associated plasmid transmission. Despite applying liberal thresholds to increase sensitivity, we were only able to detect transmission events with direct epidemiological and genomic links in 8 (16%) of 49 patients. The absence of spatiotemporal overlap but high genomic relatedness of plasmids may be explained by undetected acquisition in the hospital such as from asymptomatic NDM colonized patients. We hypothesize that this is the most likely explanation for our findings because we did not conduct routine prospective surveillance. Prior work has shown that relying on clinical disease for detection substantially underestimates the number of patients colonized with carbapenemase-producing organisms.^15,31^ In addition, environmental sources (eg, hospital sinks and drains) are increasingly recognized as reservoirs of carbapenemase-producing organisms.^32^ We assessed for sink colonization in the ICU in 2019 and did not detect NDM-carrying organisms,^33,34^ but we have not repeated systematic testing subsequently. Acquisition outside our institution (eg, in the community or in other hospital networks) is also possible. Increased local NDM acquisition in the Australian state of Victoria has been highlighted as a growing problem in recent state guidelines.^13^ Indeed, in the 3 months prior, 67% of patients had a history of admission to hospital and 31% had a history of admission to the ICU. Our findings raise concern about a potential widespread NDM outbreak with subsequent establishment of endemicity, as noted due to healthcare-associated transmission across multiple institutions in Tuscany in 2018–2019.^5,35–37^

Through our comprehensive long- and short-read sequencing of all study isolates, we were able to unravel several aspects of NDM plasmid spread. First, interstrain transfer of NDM plasmids occurred commonly and was a key driver for the increased NDM case numbers in 2019–2021. In addition, we demonstrated that interstrain transfer likely occurred within patients, highlighting the potential for diversification of genetic settings of epidemic plasmids within patients’ microbiomes.^33,38^ Second, although sophisticated plasmid clustering techniques provided much higher resolution than approaches such as *rep* typing, NDM plasmid plasticity rendered even these clusters imperfect.^24^ We noted closely related plasmid backbones that carried different NDM variants (eg, IncX3 plasmids) or NDM flanking regions. Finally, in addition to conducting plasmid-level analyses, we showed that NDM spread may result from mobile genetic elements smaller than plasmids, such as the NDM transposon we identified in multiple plasmid backbones, including within the same patient. This transposon was also noted as one of the most common NDM flanking regions in a recent comprehensive analysis of global NDM plasmids.^30^

These findings have important implications for use of WGS for outbreak detection where horizontal spread is the major contributor. Although determining whether plasmids are likely identical is feasible (eg, our analyses of IncN and IncX3 epidemic plasmids), defining differences between plasmids is a more complex endeavor. An objective threshold of plasmid relatedness suggestive of transmission is needed and will likely require use of multiple metrics including measures of identity (eg, SNV analyses, average nucleotide identity), gene content, and large-scale structural rearrangements and may vary between different plasmids.^39^ Circular, closed plasmid assemblies enabled by long-read sequencing will be central to these efforts.^40^

This study had several limitations. First, the research was observational and was based at a single center. Although we noted a diverse range of NDM plasmids and variants, these findings may not be generalizable to all NDMs. Second, we only conducted active surveillance in selected patients, likely limiting our detection of NDM colonization. However, we did have systematic collection of all clinical NDM isolates with resulting accurate detection of NDM infection episodes. Finally, we used assembly approaches that result in the highest-quality plasmid assemblies, but plasmid assembly remains challenging and necessitates manual review and curation.

In summary, our study determined the changing dynamics of NDM spread in our institution as the epidemiology shifted from an association with travel to likely local acquisition. Using long-read sequencing, we were able to pinpoint this to the arrival of successful epidemic NDM IncN and IncX3 plasmids that circulated concurrently and were noted in many bacterial hosts, suggesting interstrain transfer. These epidemic plasmids remained remarkably stable across multiple bacterial strains within and between patients. We also noted that plasmids were capable of substantial plasticity, complicating efforts at determining plasmid transmission. These findings have important implications for the future use of long-read sequencing in detection and control of outbreaks where horizontal transmission plays a significant role.

## Supporting information

Macesic et al. supplementary material Macesic et al. supplementary material

## Supplementary material

To view supplementary material for this article, please visit https://doi.org/10.1017/ice.2024.8
