# Supplementary material for: Genomic investigation of multispecies and multivariant blaNDM outbreak reveals key role of horizontal plasmid transmission
Source: Infect Control Hosp Epidemiol. 2024 Feb 12;45(6):709–16. doi: 10.1017/ice.2024.8 (PMC11102827; doi:10.1017/ice.2024.8)
Supplement: Macesic et al. supplementary material [file S0899823X24000084sup001.pdf]

Supplementary Methods

Culture, DNA extraction and sequencing

All bacterial isolates were grown on cation-adjusted Mueller-Hinton II agar (Becton-Dickinson) at 37°C for 16 hours. Genomic DNA was extracted from bacterial plate culture using the GenFind V3 Reagent Kit (Beckman Coulter) as per manufacturer's instructions. Libraries for short read sequencing were prepared using the Nextera Flex DNA Library Prep Kit (Illumina), and 150 bp paired-end sequencing was performed on the NovaSeq 6000 system (Illumina). Libraries for long-read sequencing were prepared using the Ligation Sequencing Kit with Native Barcoding Expansion (Oxford Nanopore Technologies) and sequenced on the MinION instrument with an R9.4.1 flow cell (Oxford Nanopore Technologies) for 48 hours. Basecalling was performed with Guppy v.4.0.14 using the 'high accuracy' basecalling model.

De novo assembly and annotation

We constructed *de novo* assemblies incorporating short- and long-read data for all isolates. We used a long-read-first assembly approach using a bespoke pipeline (https://github.com/HughCottingham/clinopore-nf) that incorporates Flye v2.9.2 with subsequent polishing with Medaka v1.8.0, Polypolish v0.5.0 and Polca v3.4.1 [1-5]. Plasmid circularisation was assessed using Flye output. If *bla*$_{NDM}$ contigs were non-circularised, we re-assembled genomes using Unicycler v.0.4.08 with standard parameters [6]. Assembly quality was checked using Quast v5.2.0 [7] and species identification was performed using GTDB-Tk v1.0.2 [8] and checked against isolate identification performed at time of isolate collection. Genomes were annotated using Prokka v1.14.6 [9]. We then performed resistance gene and plasmid replicon detection with Abricate v.1.0.0 [10], using the NCBI Antibiotic Resistance and PlasmidFinder databases, respectively. We determined *in silico* multi-locus sequence type (ST) using 'mlst' v.2.19.0 [11]. All inconclusive ST calls with 'mlst' were checked with SRST2 v0.2.0 [12].

Core genome-based phylogenetic analyses

We performed core genome-based phylogenetic analyses on key STs, defined as those with ≥2 genomes available from ≥2 patients to create a core genome alignment. RefSeq genomes of the same ST were included for context in phylogenetic analyses. We chose one completed, closed

assembly from our institution for each ST to use as a reference. Mobile genetic elements were excluded from these reference assemblies using PHASTER and IslandViewer 4 [13, 14]. A core chromosomal single nucleotide variant (SNV) alignment was generated using Snippy v.4.6.0 [15] and recombination was removed using Gubbins v3.3 [16]. We then used this core genome alignment in IQtree v.2.0.3 to generate maximum likelihood phylogenies for each ST [17], with the best-fit model chosen using ModelFinder [18]. For each ST, median SNV distances between isolates from our institution were then calculated.

Plasmid analyses

Using Abricate, we identified bla_{NDM}-harboring contigs that were putative plasmids in our hybrid assemblies. We then used the MOB-typer v1.4.9 tool to determine plasmid replicons present, as well as to assign clusters [19]. We identified possible mosaic plasmids resulting from fusion events by examining plasmid replicon content within MOB-typer cluster and identifying plasmids which had presence of additional plasmid replicons then manually inspecting the assemblies.

We then conducted analyses within all plasmid groups within our dataset with >1 plasmid. In order to identify SNVs in the plasmid backbone, we used Snippy v.4.6.0 [15] to create a core SNV alignment by mapping short reads to a reference plasmid from our institution from each plasmid group. We used progressiveMauve v2.4.0.r4736 to align all plasmids within a plasmid group and assess for structural re-arrangements [20].

We used Flanker v0.1.5 [21] to identify and cluster flanking sequences around bla_{NDM}. We performed clustering 5000 bp downstream of the bla_{NDM} gene across windows in 500 bp increments. Clustering was only performed downstream due to prior findings from Acman et al. which showed very high diversity of flanking regions upstream of bla_{NDM} [22]. We extracted flanking regions and performed progressiveMauve to align all flanking sequences. Geneious v10.2.6 (https://www.geneious.com) was used to visualize and assess for structural re-arrangements, with subsequent manual annotation in Adobe Illustrator v2020.24.3. We compared key plasmid groups (IncX3, IncN and IncC) from the Alfred Hospital by using a representative plasmid from each group as a reference and conducting

BLAST searches and noting the top 5 matches. For the best NDM plasmid matches, we visualized the comparisons between Alfred Hospital and publicly available plasmids in Easyfig v2.2.6 [23].

Data availability

Illumina/Nanopore read data were deposited in the NCBI Sequence Read Archive under project accession PRJNA967113. Completed genome assemblies were deposited in GenBank; accessions are listed in Supp. Table 1.

Code availability

The code generated during this study is available on GitHub (https://github.com/nenadmacesic/alfred_ndm).

References

1. Wick RR, Schultz MB, Zobel J, Holt KE. Bandage: interactive visualization of de novo genome assemblies. Bioinformatics **2015**; 31(20): 3350-2.
2. Kolmogorov M, Yuan J, Lin Y, Pevzner PA. Assembly of long, error-prone reads using repeat graphs. Nat Biotechnol **2019**; 37(5): 540-6.
3. Wick RR, Holt KE. Polypolish: Short-read polishing of long-read bacterial genome assemblies. PLoS Comput Biol **2022**; 18(1): e1009802.
4. Wick RR, Judd LM, Holt KE. Assembling the perfect bacterial genome using Oxford Nanopore and Illumina sequencing. SciELO Preprints **2022**.
5. Zimin AV, Salzberg SL. The genome polishing tool POLCA makes fast and accurate corrections in genome assemblies. PLoS Comput Biol **2020**; 16(6): e1007981.
6. Wick RR, Judd LM, Gorrie CL, Holt KE. Unicycler: Resolving bacterial genome assemblies from short and long sequencing reads. PLoS Comput Biol **2017**; 13(6): e1005595.
7. Gurevich A, Saveliev V, Vyahhi N, Tesler G. QUAST: quality assessment tool for genome assemblies. Bioinformatics **2013**; 29(8): 1072-5.
8. Chaumeil PA, Mussig AJ, Hugenholtz P, Parks DH. GTDB-Tk: a toolkit to classify genomes with the Genome Taxonomy Database. Bioinformatics **2019**; 36(6): 1925-7.
9. Seemann T. Prokka: rapid prokaryotic genome annotation. Bioinformatics **2014**; 30(14): 2068-9.
10. Seemann T. Abricate: mass screening of contigs for antimicrobial resistance or virulence genes. **2020**.
11. Seemann T. mlst: Scan contig files against traditional PubMLST typing schemes. **2020**.
12. Inouye M, Dashnow H, Raven LA, et al. SRST2: Rapid genomic surveillance for public health and hospital microbiology labs. Genome Med **2014**; 6(11): 90.
13. Arndt D, Grant JR, Marcu A, et al. PHASTER: a better, faster version of the PHAST phage search tool. Nucleic Acids Res **2016**; 44(W1): W16-21.
14. Bertelli C, Laird MR, Williams KP, et al. IslandViewer 4: expanded prediction of genomic islands for larger-scale datasets. Nucleic Acids Res **2017**; 45(W1): W30-W5.
15. Seemann T. Snippy: Rapid haploid variant calling and core SNP phylogeny. Available at: https://github.com/tseemann/snippy. Accessed October 11, 2020.
16. Croucher NJ, Page AJ, Connor TR, et al. Rapid phylogenetic analysis of large samples of recombinant bacterial whole genome sequences using Gubbins. Nucleic Acids Res **2015**; 43(3): e15.
17. Minh BQ, Schmidt HA, Chernomor O, et al. IQ-TREE 2: New Models and Efficient Methods for Phylogenetic Inference in the Genomic Era. Mol Biol Evol **2020**; 37(5): 1530-4.
18. Kalyaanamoorthy S, Minh BQ, Wong TKF, von Haeseler A, Jermiin LS. ModelFinder: fast model selection for accurate phylogenetic estimates. Nat Methods **2017**; 14(6): 587-9.
19. Robertson J, Bessonov K, Schonfeld J, Nash JHE. Universal whole-sequence-based plasmid typing and its utility to prediction of host range and epidemiological surveillance. Microb Genom **2020**; 6(10).
20. Darling AE, Mau B, Perna NT. progressiveMauve: multiple genome alignment with gene gain, loss and rearrangement. Plos One **2010**; 5(6): e11147.
21. Matlock W, Lipworth S, Constantinides B, et al. Flanker: a tool for comparative genomics of gene flanking regions. Microb Genom **2021**; 7(9).
22. Acman M, Wang R, van Dorp L, et al. Role of mobile genetic elements in the global dissemination of the carbapenem resistance gene bla(NDM). Nat Commun **2022**; 13(1): 1131.
23. Sullivan MJ, Petty NK, Beatson SA. Easyfig: a genome comparison visualizer. Bioinformatics **2011**; 27(7): 1009-10.

Supp. Table 1 - Summary of study isolates

Patient ID	Isolate	Species	MLST	Specimen type	Assembly type	Collection date	NDM variant	Other carbapenemases present	NDM genetic setting	Illumina SRA accession no.	Oxford Nanopore SRA accession no.	GenBank accession no.
1	CPO053	*Enterobacter hormaechei*	114	Rectal swab	Unicycler	10/12/2018	blaNDM-7	blaIMP-4	IncX3 (AA038_AH615)	SRR23384660	SRR23384664	Pending
2	CPO438	*Escherichia coli*	967	Swab	Unicycler	31/12/2020	blaNDM-1		IncC (AA860_AJ272)	SRR24474878	SRR24474916	Pending
3	CPO340	*Klebsiella quasipneumoniae*	5551	Rectal swab	Flye	28/4/2020	blaNDM-1		IncN (AA552_AI757)	SRR24474933	SRR24474971	Pending
3	CPO336	*Escherichia coli*	131	Rectal swab	Unicycler	16/5/2020	blaNDM-1		IncN (AA552_AI757)	SRR24474934	SRR24474972	Pending
4	CPO219	*Escherichia coli*	405	Rectal swab	Flye	2/4/2019	blaNDM-5		IncFIA (AA170_AH818)	SRR24474958	SRR24475024	Pending
5	CPO195	*Escherichia coli*	361	Urine	Unicycler	27/2/2019	blaNDM-5		IncX3 (AA038_AH615)	SRR24474963	SRR24474999	Pending
6	CPO036	*Klebsiella pneumoniae*	16	Rectal swab	Flye	10/1/2019	blaNDM-5		IncX3 (AA038_AH615)	SRR24475026	SRR24475015	Pending
6	CPO035	*Escherichia coli*	8346	Rectal swab	Flye	10/1/2019	blaNDM-5		IncFIA (AA170_AH820)	SRR24475027	SRR24475016	Pending
7	CPO319	*Klebsiella pneumoniae*	16	Urine	Unicycler	21/2/2020	blaNDM-1		IncFIB (AA405_AI436)	SRR24474942	SRR24474980	Pending
8	CPO371	*Citrobacter freundii*	492	Rectal swab	Unicycler	18/6/2020	blaNDM-7	blaIMP-4	IncX3 (AA038_AH615)	SRR23384825	SRR23384576	Pending
8	CPO434	*Klebsiella pneumoniae*	17	Urine	Flye	14/11/2020	blaNDM-7		IncX3 (AA038_AH615)	SRR24474880	SRR24474918	Pending
8	CPO408	*Klebsiella pneumoniae*	17	Rectal swab	Flye	18/6/2020	blaNDM-7	blaIMP-4	IncX3 (AA038_AH615)	SRR24474885	SRR24474923	Pending
9	CPO429	*Klebsiella aerogenes*	304	Urine	Flye	8/10/2020	blaNDM-1	blaIMP-4	IncN (AA552_AI757)	SRR23384815	SRR23384532	Pending
10	CPO363	*Klebsiella quasipneumoniae*	5551	Peritoneal fluid	Flye	7/6/2020	blaNDM-1		IncN (AA552_AI757)	SRR24474893	SRR24474931	Pending
11	CPO391	*Enterobacter hormaechei*	1015	Urine	Flye	10/7/2020	blaNDM-1		IncC (AA860_AJ272)	SRR24474888	SRR24474926	Pending
11	CPO390	*Enterobacter hormaechei*	1015	Rectal swab	Flye	7/7/2020	blaNDM-1		IncC (AA860_AJ272)	SRR24474889	SRR24474927	Pending
12	CPO299	*Enterobacter hormaechei*	88	Rectal swab	Flye	11/12/2019	blaNDM-1		IncN (AA552_AI757)	SRR24474950	SRR24474988	Pending

13	CPO084	*Escherichia coli*	405	Rectal swab	Flye	20/4/2018	blaNDM-5		IncFIA (AA170_AH818)	SRR24474883	SRR24475010	Pending
13	CPO083	*Escherichia coli*	405	Urine	Flye	25/4/2018	blaNDM-5		IncFIA (AA170_AH818)	SRR24474894	SRR24475011	Pending
14	CPO592	*Klebsiella pneumoniae*	485	Urine	Flye	20/6/2021	blaNDM-1		IncN (AA552_AI757)	SRR24475020	SRR24474902	Pending
14	CPO591	*Klebsiella pneumoniae*	485	Rectal swab	Flye	9/6/2021	blaNDM-1		IncN (AA552_AI757)	SRR24475021	SRR24474903	Pending
15	CPO236	*Escherichia coli*	176	Rectal swab	Flye	28/5/2019	blaNDM-1		IncC (AA860_AJ272)	SRR24474954	SRR24474992	Pending
16	CPO350	*Citrobacter freundii*	64	Rectal swab	Flye	20/5/2020	blaNDM-1		IncN (AA552_AI757)	SRR24474899	SRR24474969	Pending
17	CPO070	*Escherichia coli*	1284	Rectal swab	Flye	3/9/2018	blaNDM-5		IncX3 (AA038_AH615)	SRR24474959	SRR24475014	Pending
18	CPO404	*Klebsiella michiganensis*	85_2LV	Rectal swab	Flye	29/7/2020	blaNDM-1		IncN (AA552_AI757)	SRR24474887	SRR24474925	Pending
19	CPO124	*Escherichia coli*	405	Urine	Flye	7/9/2016	blaNDM-5		IncFIA (AA170_AH818)	SRR24474964	SRR24475002	Pending
20	CPO097	*Escherichia coli*	648	Rectal swab	Unicycler	15/12/2017	blaNDM-7		IncX3 (AA038_AH615)	SRR24475006	SRR24475007	Pending
21	CPO090	*Escherichia coli*	410	Rectal swab	Flye	25/2/2018	blaNDM-5		IncFIA (AA323_AI214)	SRR24474872	SRR24475009	Pending
22	CPO096	*Escherichia coli*	46	Rectal swab	Flye	16/12/2017	blaNDM-5		IncFIA (AA172_AH832)	SRR24475017	SRR24475008	Pending
23	CPO316	*Enterobacter cloacae*	610	Rectal swab	Flye	17/2/2020	blaNDM-1		IncN (AA552_AI757)	SRR24474944	SRR24474982	Pending
24	CPO126	*Klebsiella pneumoniae*	14	Rectal swab	Flye	7/9/2016	blaNDM-1		IncFIB (AA018_AH560)	SRR24474910	SRR24475000	Pending
24	CPO125	*Escherichia coli*	410	Rectal swab	Flye	7/9/2016	blaNDM-5		IncFIA (AA323_AI214)	SRR24474921	SRR24475001	Pending
24	CPO122	*Escherichia coli*	410	Urine	Flye	30/10/2016	blaNDM-5	blaOXA-181	IncFIA (AA323_AI214)	SRR24474975	SRR24475003	Pending
25	CPO114	*Escherichia coli*	394	Urine	Flye	5/1/2017	blaNDM-1		IncFIA (AA327_AI227) | Chromosome	SRR24474986	SRR24475004	Pending
26	CPO433	*Klebsiella michiganensis*	85_2LV	Rectal swab	Unicycler	12/11/2020	blaNDM-1		IncN (AA552_AI757)	SRR24474881	SRR24474919	Pending
27	CPO230	*Escherichia coli*	90	Rectal swab	Flye	16/5/2019	blaNDM-5		IncFIA (AA170_AH818)	SRR24474955	SRR24474993	Pending

28	CPO406	*Citrobacter braakii*	Novel	Rectal swab	Unicycler	24/5/2020	blaNDM-7		IncX3 (AA038_AH615)	SRR24474886	SRR24474924	Pending
28	CPO354	*Klebsiella pneumoniae*	17	Rectal swab	Unicycler	24/5/2020	blaNDM-7		IncX3 (AA038_AH615)	SRR24474896	SRR24474966	Pending
29	CPO334	*Klebsiella quasipneumoniae*	5551	Urine	Flye	28/4/2020	blaNDM-1		IncN (AA552_AI757)	SRR24474935	SRR24474973	Pending
29	CPO331	*Klebsiella quasipneumoniae*	5551	Rectal swab	Flye	24/4/2020	blaNDM-1		IncN (AA552_AI757)	SRR24474936	SRR24474974	Pending
29	CPO330	*Klebsiella quasipneumoniae*	5551	Faeces	Flye	21/4/2020	blaNDM-1		IncN (AA552_AI757)	SRR24474938	SRR24474976	Pending
29	CPO329	*Citrobacter amaloticus*	-	Faeces	Flye	21/4/2020	blaNDM-1		IncN (AA552_AI757)	SRR24474939	SRR24474977	Pending
29	CPO326	*Klebsiella quasipneumoniae*	5551	Sputum	Flye	12/3/2020	blaNDM-1		IncN (AA552_AI757)	SRR24474941	SRR24474979	Pending
29	CPO311	*Citrobacter amaloticus*	-	Rectal swab	Flye	29/1/2020	blaNDM-1		IncN (AA552_AI757)	SRR24474946	SRR24474984	Pending
29	CPO310	*Klebsiella quasipneumoniae*	5551	Rectal swab	Flye	29/1/2020	blaNDM-1		IncN (AA552_AI757)	SRR24474947	SRR24474985	Pending
30	CPO099	*Escherichia coli*	405	Rectal swab	Flye	7/11/2017	blaNDM-5		IncFIA (AA324_AI219)	SRR24475025	SRR24475005	Pending
31	CPO080	*Escherichia coli*	405	Rectal swab	Flye	9/5/2018	blaNDM-5		IncI-gamma/K1 (AC026_AL043)	SRR24474937	SRR24475012	Pending
31	CPO077	*Escherichia coli*	405	Urine	Flye	13/5/2018	blaNDM-5		IncI-gamma/K1 (AC026_AL043)	SRR24474948	SRR24475013	Pending
32	CPO351	*Enterobacter hormaechei*	1015	Rectal swab	Flye	20/5/2020	blaNDM-1		IncC (AA860_AJ272)	SRR24474898	SRR24474968	Pending
33	CPO218	*Klebsiella pneumoniae*	15	Blood Peripheral	Flye	1/4/2019	blaNDM-4		IncFII (AA450_AI539)	SRR24474960	SRR24474996	Pending
33	CPO217	*Klebsiella pneumoniae*	15	Urine	Flye	19/3/2019	blaNDM-4		IncFII (AA450_AI539)	SRR24474961	SRR24474997	Pending
33	CPO206	*Klebsiella pneumoniae*	656	Rectal swab	Flye	15/3/2019	blaNDM-4		IncFII (AA450_AI539)	SRR24474962	SRR24474998	Pending
34	CPO225	*Enterobacter hormaechei*	114	Rectal swab	Unicycler	15/4/2019	blaNDM-7		IncX3 (AA038_AH615)	SRR24474957	SRR24474995	Pending
35	CPO228	*Acinetobacter baumannii*	2	Wound	Flye	4/5/2019	blaNDM-1	blaOXA-23 | blaOXA-66	Chromosome	SRR24474956	SRR24474994	Pending
36	CPO293	*Klebsiella variicola*	596	Wound	Unicycler	30/11/2019	blaNDM-1		IncN (AA552_AI757)	SRR24474951	SRR24474989	Pending
36	CPO282	*Klebsiella michiganensis*	Novel	Rectal swab	Flye	6/11/2019	blaNDM-1		IncN (AA552_AI757)	SRR24474952	SRR24474990	Pending

36	CPO266	*Klebsiella michiganensis*	Novel	Rectal swab	Unicycler	6/11/2019	blaNDM-1		IncN (AA552_AI757)	SRR24474953	SRR24474991	Pending
37	CPO450	*Citrobacter braakii*	567	Rectal swab	Flye	9/2/2021	blaNDM-7		IncX3 (AA038_AH615)	SRR24474868	SRR24474906	Pending
37	CPO449	*Escherichia coli*	127	BAL	Unicycler	29/1/2021	blaNDM-7		IncX3 (AA038_AH615)	SRR24474869	SRR24474907	Pending
37	CPO446	*Escherichia coli*	127	Pleural fluid	Unicycler	29/1/2021	blaNDM-7		IncX3 (AA038_AH615)	SRR24474873	SRR24474911	Pending
38	CPO453	*Klebsiella michiganensis*	Novel	Rectal swab	Flye	26/2/2021	blaNDM-1		IncN (AA552_AI757)	SRR24475022	SRR24474904	Pending
38	CPO452	*Citrobacter youngae*	187	Rectal swab	Flye	26/2/2021	blaNDM-1		IncN (AA552_AI757)	SRR24475023	SRR24474905	Pending
39	CPO308	*Escherichia coli*	648	Rectal swab	Unicycler	17/1/2020	blaNDM-5		IncX3 (AA038_AH615)	SRR24474949	SRR24474987	Pending
40	CPO358	*Klebsiella pneumoniae*	16	Urine	Flye	10/6/2020	blaNDM-1		IncFIB (AA405_AI436)	SRR24474895	SRR24474965	Pending
40	CPO352	*Klebsiella pneumoniae*	16	Swab	Flye	20/5/2020	blaNDM-1		IncFIB (AA405_AI436)	SRR24474897	SRR24474967	Pending
40	CPO327	*Klebsiella pneumoniae*	11	Wound	Flye	13/3/2020	blaNDM-5	blaOXA-48	IncFIB (AA405_AI436)	SRR24474940	SRR24474978	Pending
40	CPO318	*Klebsiella pneumoniae*	16	Rectal swab	Unicycler	19/2/2020	blaNDM-1		IncFIB (AA405_AI436)	SRR24474943	SRR24474981	Pending
40	CPO315	*Klebsiella pneumoniae*	16	Wound	Flye	16/2/2020	blaNDM-1		IncFIB (AA405_AI436)	SRR24474945	SRR24474983	Pending
41	CPO443	*Pseudomos aeruginosa*	644	Faeces	Flye	12/3/2020	blaNDM-1		Chromosome	SRR24474874	SRR24474912	Pending
41	CPO430	*Pseudomos aeruginosa*	644	Faeces	Flye	12/3/2020	blaNDM-1	blaIMP-62	Chromosome	SRR24474882	SRR24474920	Pending
41	CPO384	*Pseudomos aeruginosa*	644	Faeces	Flye	12/3/2020	blaNDM-1	blaIMP-62	Chromosome	SRR24474891	SRR24474929	Pending
41	CPO383	*Pseudomos aeruginosa*	644	Burn	Flye	2/3/2020	blaNDM-1	blaIMP-62	Chromosome	SRR24474892	SRR24474930	Pending
42	CPO346	*Raoultella ornithinolytica*	-	Rectal swab	Flye	15/5/2020	blaNDM-1		IncN (AA552_AI757)	SRR24474932	SRR24474970	Pending
43	CPO387	*Escherichia coli*	940_1LV	Swab	Flye	29/6/2020	blaNDM-5		IncX3 (AA038_AH615)	SRR24474890	SRR24474928	Pending
44	CPO448	*Enterobacter hormaechei*	1015	Rectal swab	Flye	1/2/2021	blaNDM-1		IncC (AA860_AJ272)	SRR24474870	SRR24474908	Pending
44	CPO447	*Enterobacter hormaechei*	1015	Swab	Flye	29/1/2021	blaNDM-1		IncC (AA860_AJ272)	SRR24474871	SRR24474909	Pending

45	CPO428	*Citrobacter braakii*	567	Tissue	Flye	28/9/2020	blaNDM-7		IncX3 (AA038_AH615)	SRR24474884	SRR24474922	Pending
46	CPO436	*Enterobacter hormaechei*	1015	Rectal swab	Flye	20/12/2020	blaNDM-1		IncC (AA860_AJ272)	SRR24474879	SRR24474917	Pending
47	CPO595	*Klebsiella michiganensis*	29	Urine	Flye	28/6/2021	blaNDM-1		IncN (AA552_AI757)	SRR24475018	SRR24474900	Pending
48	CPO442	*Klebsiella pneumoniae*	16	Rectal swab	Flye	5/1/2021	blaNDM-1	blaOXA-232	IncFIA (AA170_AH820)	SRR24474875	SRR24474913	Pending
48	CPO441	*Escherichia coli*	1193	Rectal swab	Unicycler	5/1/2021	blaNDM-1		IncC (AA860_AJ272)	SRR24474876	SRR24474914	Pending
48	CPO440	*Escherichia coli*	1193	Rectal swab	Unicycler	1/1/2021	blaNDM-1		IncC (AA860_AJ272)	SRR24474877	SRR24474915	Pending
49	CPO593	*Escherichia coli*	1284	Rectal swab	Flye	18/6/2021	blaNDM-5		IncFIA (AA324_AI219)	SRR24475019	SRR24474901	Pending

Abbreviations: ID - identification, LV - locus variant, MLST - Multi-locus sequence type, NDM - New Delhi metallo-beta-lactamase, SRA - Sequence Read Archive.

Supp. Table 2 – Pairwise SNV distances of key New Delhi metallo-beta-lactamase bacterial host strains

Bacterial strain	Total genomes	Median pairwise SNV distance	Interquartile range	Total patients	Minimum between-patient pairwise SNV distance
Escherichia coli ST405	7	121	103 – 300	5	32
Klebsiella pneumoniae ST16	7	134.5	4 – 171.25	4	0
Klebsiella quasipneumoniae ST5551	7	8	4 – 11	3	2
Enterobacter hormaechei ST1015	6	4	3 – 5	4	3
Escherichia coli ST410	3	78	28.75 – 110	2	110
Klebsiella michiganensis - Novel ST	3	24	12 – 24	2	24
Klebsiella pneumoniae ST17	3	2	1.5 – 2.5	2	2
Citrobacter braakii ST567	2	1*		2	1
Enterobacter hormaechei ST114	2	1847*		2	1847
Escherichia coli ST1284	2	3417*		2	3417
Escherichia coli ST648	2	11845*		2	11845
Klebsiella michiganensis ST85 (2LV)	2	4*		2	4

*Pairwise distance reported as only two genomes available for analyses

Abbreviations: LV – locus variant; SNV – single nucleotide variant; ST – sequence type

Supp. Table 3 – Pairwise SNV distances of key New Delhi metallo-beta-lactamase plasmid groups

Plasmid group (MOBtyper cluster)	Median pairwise SNV distance	Mean pairwise SNV distance	Interquartile range
IncC (AA860 AJ272)	1	8.2	0 - 22
IncFIA (AA170 AH818)	835	768.5	647.25 - 1115
IncFIA (AA170 AH820)	196*		
IncFIA (AA323 AI214)	228	152.6	114.5 - 228.5
IncFIA (AA324 AI219)	41*		
IncFIB (AA405 AI436)	0	47.7	0 - 143
IncFII (AA450 AI539)	267	178	133.5 - 267
IncI-gamma/K1 (AC026 AL043)	4*		
IncN (AA552 AI757)	0	0.08	0 - 0
IncX3 (AA038 AH615)	2	2.2	0 - 3

*Pairwise distance reported as only two genomes available for analyses

Abbreviations: SNV – single nucleotide variant

Supp. Table 4 – BLAST matches for Alfred Hospital epidemic plasmids

Reference plasmid	Description	Max Score	Query Coverage	E value	Percentage identity	GenBank accession
Alfred Hospital IncX3	*Escherichia coli* strain EC-YC1908-399 plasmid p399-4, complete sequence	85148	100%	0	99.96	CP084538.1
	Escherichia coli EC08 plasmid pEC08_NDM5 DNA, complete sequence	84987	100%	0	99.9	LC521848.1
	Escherichia coli strain CFSAN064035 plasmid pGMI17-003_4, complete sequence	84108	100%	0	99.94	CP031138.1
	Klebsiella quasipneumoniae strain JNQH473 plasmid pJNQH473-4, complete sequence	84051	100%	0	99.95	CP075887.1
	Klebsiella pneumoniae strain KpN01 plasmid pKpN01-NDM7, complete sequence	83534	100%	0	99.95	CP012990.1
Alfred Hospital IncN	*Citrobacter freundii* strain Cf7308 plasmid pNDM-Cf7308, complete sequence	53033	95%	0	99.93	CP092465.1
	Enterobacter asburiae strain 5549 plasmid IncN_1, complete sequence	53033	95%	0	99.93	CP093156.1
	Escherichia coli strain Z30 plasmid pZ30-NDM-29, complete sequence	53033	95%	0	99.93	CP066846.1
	Enterobacter cloacae strain CBG15936 plasmid pNDM1-CBG, complete sequence	53029	95%	0	99.93	CP046118.1
	Klebsiella pneumoniae strain FF1009 plasmid pFF1009_1, complete sequence	52951	91%	0	99.95	CP077825.1
Alfred Hospital IncC	*Klebsiella pneumoniae* isolate BB1465 genome assembly, plasmid: pKP-CTX-M-15_	1.77E+05	92%	0	99.95	LR822059.1

	Proteus mirabilis strain AR_0159 plasmid tig00000137, complete sequence	1.76E+05	93%	0	99.99	CP021551.1
	Citrobacter werkmanii isolate BB1459 genome assembly, plasmid: pCW-CTX-M-15B_	1.75E+05	94%	0	99.97	LR822057.1
	Escherichia coli strain Ecol_732 plasmid pEC732_IMP14, complete sequence	1.70E+05	89%	0	99.95	CP015139.1
	Klebsiella michiganensis strain BD-50-Km plasmid pBD-50-Km_VIM-1, complete sequence	1.60E+05	94%	0	99.98	CP061931.1

Supp. Table 5 – Within-patient plasmid pairwise single nucleotide variant distances

Plasmid group (MOBtyper cluster)	Total within-patient pairs	Median SNV distance	Mean SNV distance	Interquartile range	Range
bla_{NDM-1} IncC (AA860 AJ272)	3	0	0	0 - 0	0 - 0
bla_{NDM-5} IncFIA (AA323 AI214)	1	1*			
bla_{NDM-1} IncFIB (AA405 AI436)	10	0	57.2	0 – 143	0 - 143
bla_{NDM-4} IncFII (AA450 AI539)	3	267	178	133.5 - 267	0 - 267
bla_{NDM-5} IncI-gamma/K1 (AC026 AL043)	1	4*			
bla_{NDM-1} IncN (AA552 AI757)	27	0	0	0 - 0	0 - 0
bla_{NDM-7} IncX3 (AA038 AH615)	7	0	0	0 - 0	0 - 0

*Pairwise distance reported as only two genomes available for analyses

Abbreviations: SNV – single nucleotide variant

Supp. Figure 1- Epidemiological curves of *bla*NDM 2016-2021

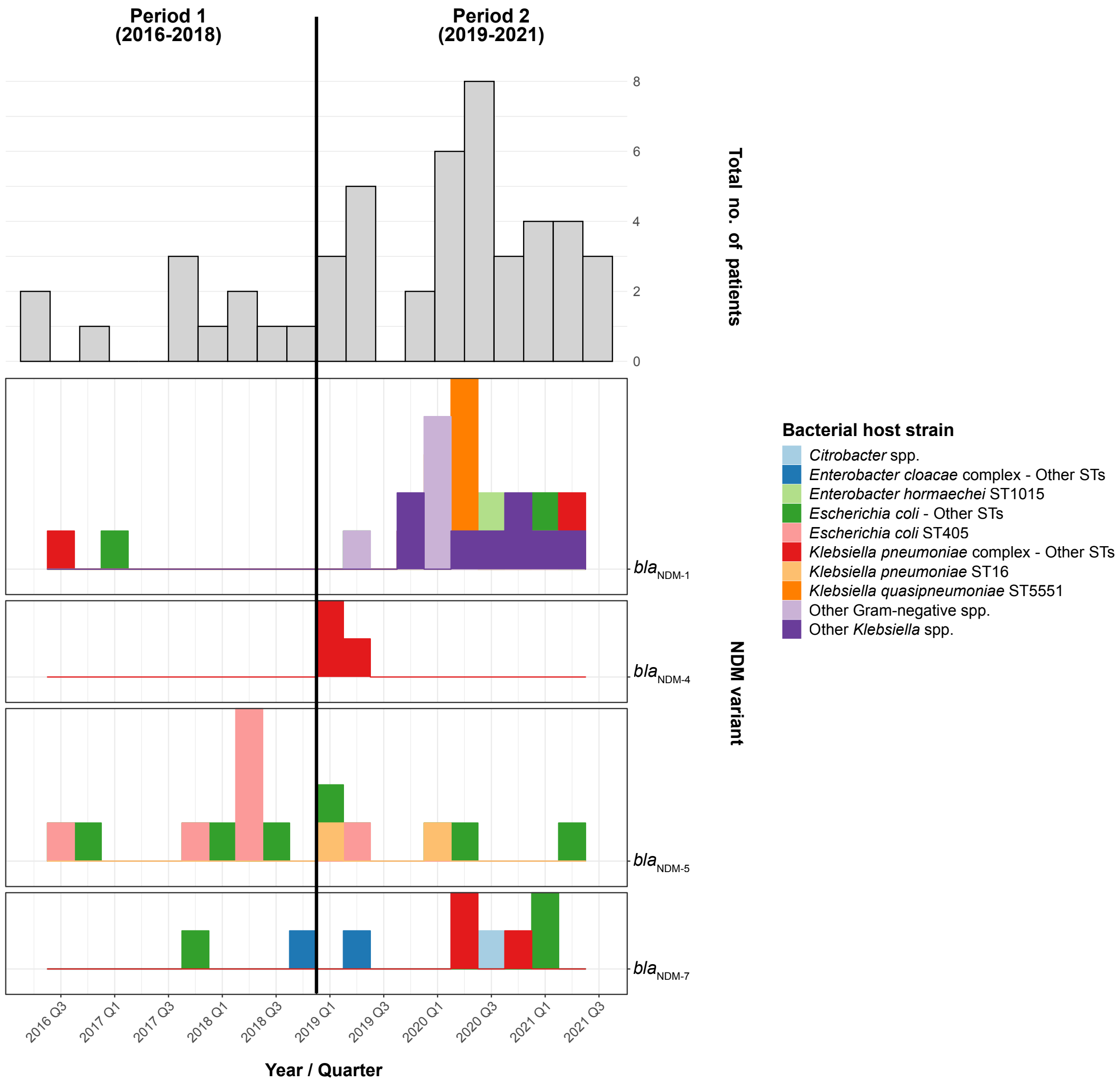

Top panel indicates overall number of NDM patients during each year/quarter. Bottom panels show *bla*NDM genomes per NDM variant (shown as separate panels) and bacterial host strain (shown as different colours).

Abbreviations: NDM – New Delhi metallo-beta-lactamase; No. – number; ST – sequence type.

Supp. Figure 2 – Comparison of Alfred Hospital and global New Delhi metallo-beta-lactamase plasmids

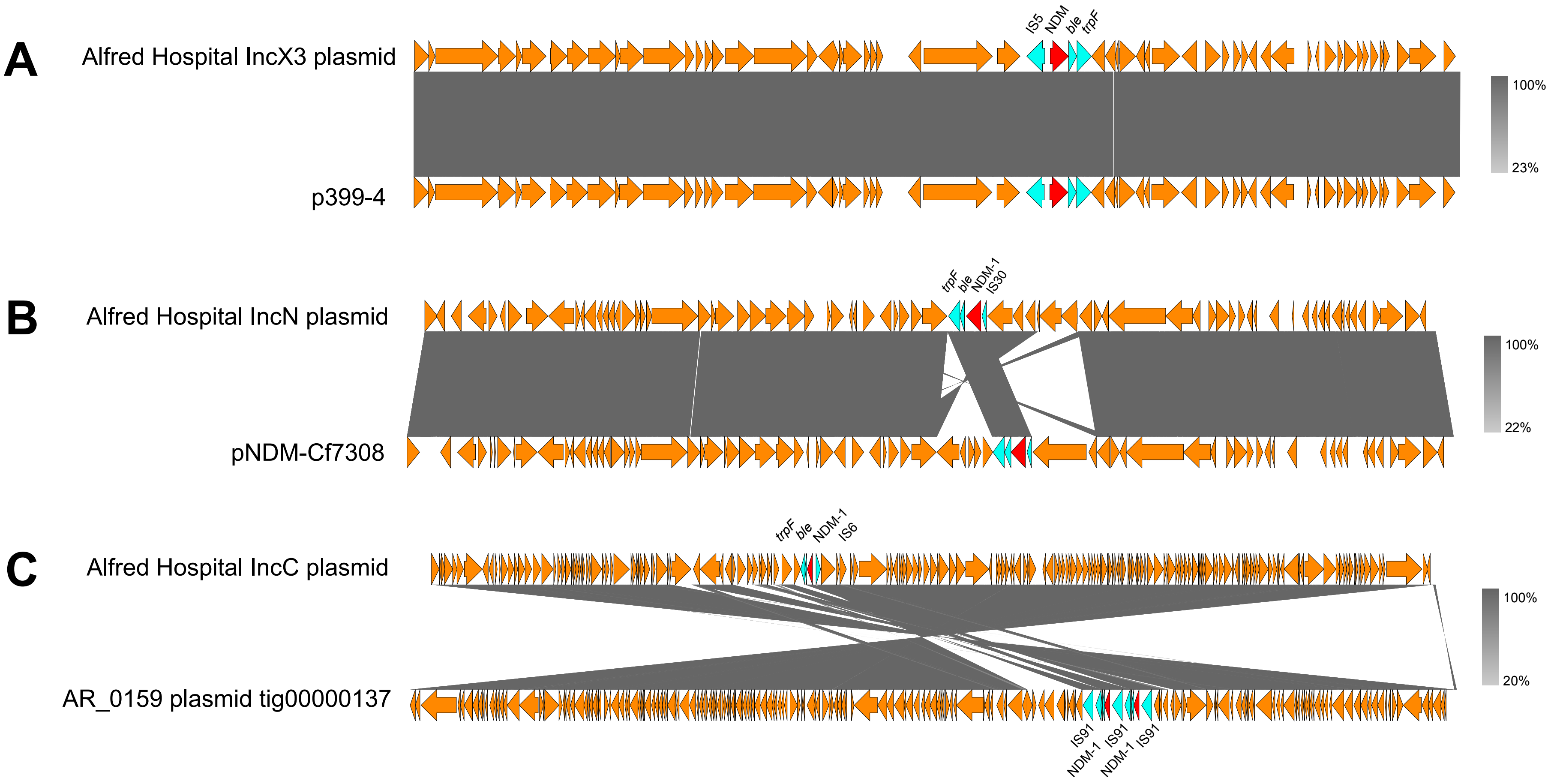

We conducted comparative analyses with global New Delhi metallo-beta-lactamase (NDM) plasmids that were closely related to NDM epidemic plasmids from our study. The location of *bla*$_{NDM}$ genes is shown in red, with the genetic context in light blue. Panel A shows that IncX3 plasmids from our study were almost identical to global NDM IncX3 plasmids (100% coverage and 99.96% identity). Panels B and C demonstrate IncN and IncC plasmids, respectively. Plasmids from our study had more significant differences to NDM global plasmids, with IncN plasmids having 95% coverage and 99.93% identity while IncC plasmids had 93% coverage and 99.99% identity.

Supp. Figure 3 – Comparative analysis of non-epidemic NDM plasmids

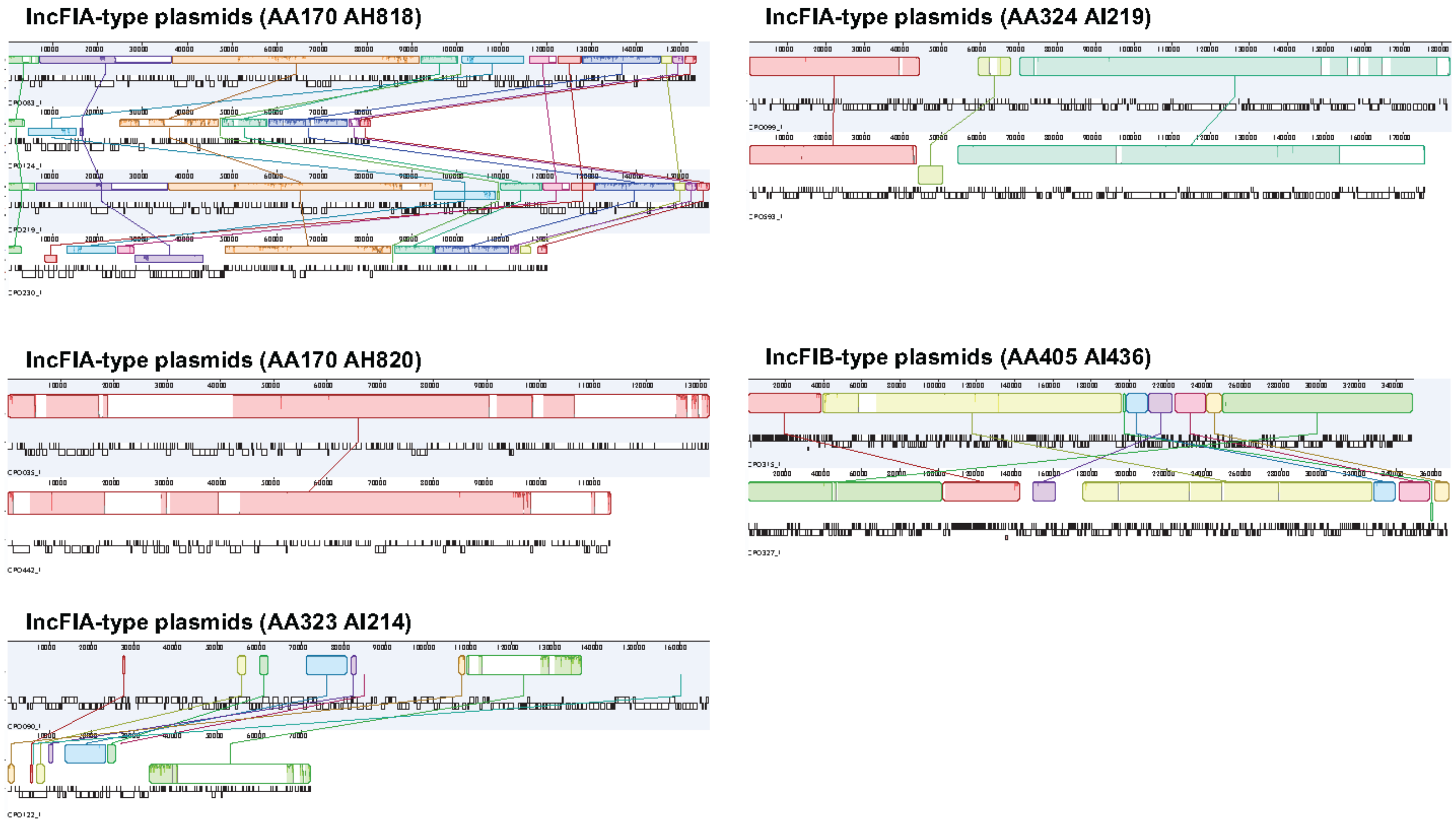

We conducted comparative analysis of non-epidemic NDM plasmid groups from our study from which multiple plasmids were available for analysis. Each colored field represents a locally collinear block, a homologous region of sequence shared by multiple plasmids without any rearrangement of that region. Same colours indicate the same regions present in different plasmids. In contast to epidemic plasmids, NDM non-epidemic plasmids showed more large-scale structural differences despite plasmids being classified within the same plasmid groups.

Abbreviations: NDM – New Delhi metallo-beta-lactamase; ST – sequence type.

Supp. Figure 4 – Patients colonised with multiple NDM plasmids

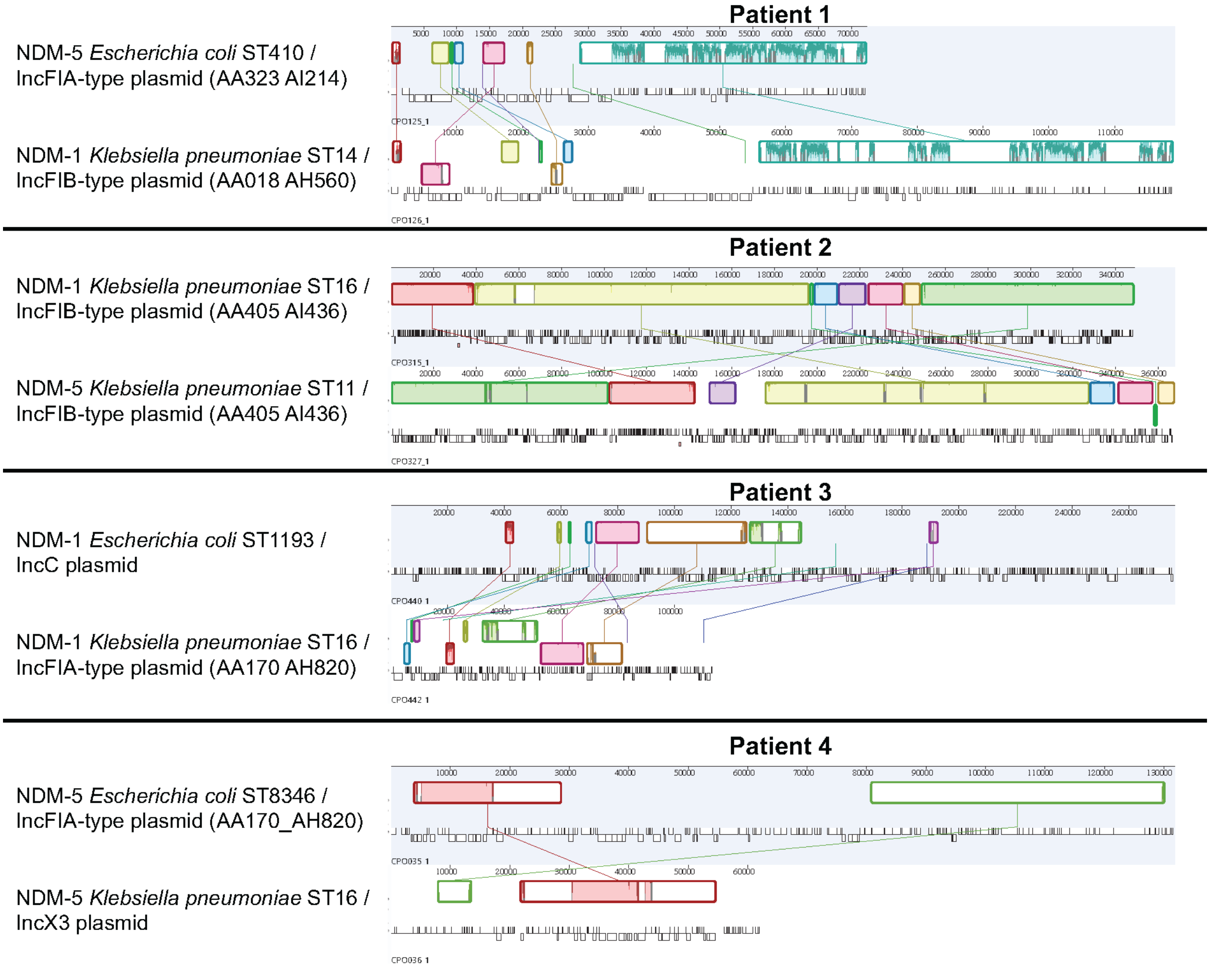

We analyzed individual patients colonised with multiple NDM plasmids as evidenced by colonisation with different NDM plasmid groups or different NDM variants. Each colored field represents a locally collinear block, a homologous region of sequence shared by multiple plasmids without any rearrangement of that region. Same colours indicate the same regions present in different plasmids.

Abbreviations: NDM – New Delhi metallo-beta-lactamase; ST – Sequence type.

Supp. Figure 5 – Analysis of potential NDM transmission events between patients

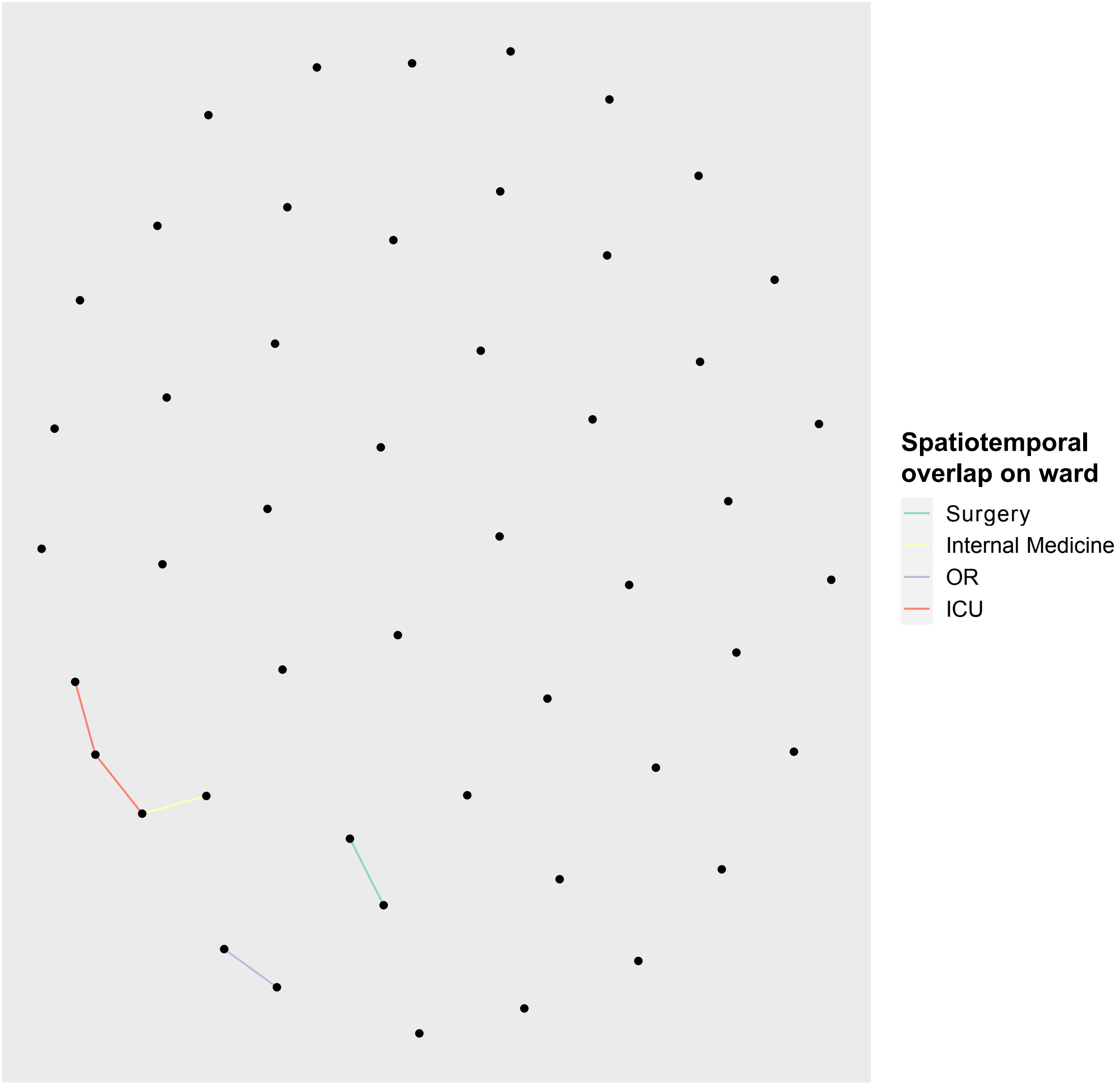

Individual patients are shown as vertices. Edges were drawn if there was spatiotemporal overlap on a hospital wards (indicated by edge colour) and a genomic criterion of presence of *bla*$_{NDM}$ in the same plasmid (defined as same plasmid group and NDM variant with ≤5 SNVs difference in the backbone).

Abbreviations: ICU – Intensive Care Unit; NDM – New Delhi metallo-beta-lactamase; OR – Operating Room.
